# Directional Coupling of Surface Plasmon Polaritons at Exceptional Points in the Visible Spectrum

**DOI:** 10.3390/ma18245595

**Published:** 2025-12-12

**Authors:** Amer Abdulghani, Salah Abdo, Khalil As’ham, Ambali Alade Odebowale, Andrey E. Miroshnichenko, Haroldo T. Hattori

**Affiliations:** School of Engineering and Technology, University of New South Wales at Canberra, Northcott Drive, Canberra, ACT 2610, Australia; s.abdo@unsw.edu.au (S.A.); k.asham@unsw.edu.au (K.A.); a.odebowale@unsw.edu.au (A.A.O.); andrey.miroshnichenko@unsw.edu.au (A.E.M.)

**Keywords:** non-hermitian photonics, surface plasmon polariton, exceptional points, guided plasmon polaritons, plasmonics

## Abstract

Robust control over the coupling and propagation of surface plasmon polaritons (SPPs) is essential for advancing various plasmonic applications. Traditional planar structures, commonly used to design SPP directional couplers, face limitations such as low extinction ratios and design complexities. These issues frequently hinder the dense integration and miniaturisation of photonic systems. Recently, exceptional points (EPs)—unique degeneracies within the parameter space of non-Hermitian systems—have garnered significant attention for enabling a range of counterintuitive phenomena in non-conservative photonic systems, including the non-trivial control of light propagation. In this work, we develop a rigorous temporal coupled-mode theory (TCMT) description of a non-Hermitian metagrating composed of alternating silicon–germanium nanostrips and use it to explore the unidirectional excitation of SPPs at EPs in the visible spectrum. Within this framework, EPs, typically associated with the coalescence of eigenvalues and eigenstates, are leveraged to manipulate light propagation in nonconservative photonic systems, facilitating the refined control of SPPs. By spatially modulating the permittivity profile at a dielectric–metal interface, we induce a passive parity–time (PT)-symmetry, which allows for refined tuning of the SPPs’ directional propagation by optimising the structure to operate at EPs. At these EPs, a unidirectional excitation of SPPs with a directional intensity extinction ratio as high as 40 dB between the left and right excited SPP modes can be reached, with potential applications in integrated optical circuits, visible communication technologies, and optical routing, where robust and flexible control of light at the nanoscale is crucial.

## 1. Introduction

The quantum mechanics formalism postulates that the Hamiltonian governing a system’s state evolution in a complex Hilbert space must be Hermitian, ensuring its eigenvalues are real [1]. This criterion guarantees that the system observables—energy measurements in this case—corresponding to these eigenvalues are real and yield physically meaningful and measurable results. This notion however has been recently challenged by Bender et al. [2], who proved that systems with non-Hermitian Hamiltonians may indeed have a real spectrum provided that such systems obey the PT-symmetry condition despite being open systems. Remarkably, these systems under non-Hermiticity parameter variation can endure spontaneous symmetry breaking and restoration upon real to complex spectral transitions at singularities called exceptional points (EPs) [3]. At exceptional points, one or more eigenstates and their corresponding eigenvalues simultaneously coalesce and become degenerate, which is notably different from the degeneracy in Hermitian systems referred to as diabolic points (DPs) [4], where only the eigenvalues coalesce. In contrast, the eigenstates remain orthogonal in the Hermitian parameter space. Hence, non-Hermitian systems exhibit reduced dimensionality at EPs [5].

Although the theoretical concept of exceptional points (EPs) originated in quantum mechanics, it sparked extensive attention in nonconservative photonic systems [6,7,8,9] and was first experimentally observed in optics and photonics [10,11]. This was due to the widespread presence of non-hermiticity in optical systems, primarily caused by intrinsic losses due to absorption and radiation leakage. Additionally, the ability to locally control the gain through methods like stimulated emission or parametric processes [12] enables easier control of non-Hermitian Hamiltonians and facilitates the exploration of exceptional points in these systems.

EPs have paved the way for fundamentally revisiting the photonic systems with losses no longer viewed as undesirable attributes but rather a useful way to manipulate the nonconservative photonic systems for novel functionalities in lasing, sensing, and light propagation control that were unattainable otherwise. It has been demonstrated that the distinctive behaviour near an EP significantly influences the spectral response and threshold of lasing cavities [13], leading to a unique reversal in pump dependence marked by a decrease in emitted light intensity as the pump power increases in coupled microdisk quantum cascade lasers [14], directional lasing in a pair of silica microcavities [15] orbital angular momentum vortex lasing [16], and single-mode lasing [17,18].

Remarkably, nanophotonic systems are increasingly recognised as versatile platforms for advanced sensing applications due to their miniaturisation capabilities and high sensitivity to small input changes [19]. In particular, it has been shown that systems of higher order EPs are subjected to eigenstates split proportional to the *n*th root of the input perturbation, where n is the EP order; hence, sensors operating at EPs are envisaged to have a superior response to perturbations compared to those at conventional degeneracies exhibiting a linear response [3,20,21], promising enhanced performance in applications ranging from biochemistry to the Internet of Things. In this context, Chen et al. [22] demonstrated nano-object detection by perturbing a micro-toroid cavity tuned to an exceptional point with two scatterers. Furthermore, photonic systems operating at an EP have been proposed for nanoparticle detection [23,24,25,26], angular rate and phase sensing [27,28], and refractive index sensing [29,30,31,32].

PT-symmetric photonic structures have also been employed to achieve non-trivial control over light propagation at exceptional points (EPs), including unidirectional reflectionless light propagation in integrated silicon waveguide meta gratings through modulation of the dielectric permittivity [33]. Additionally, the suppression of backscattering has been successfully demonstrated in a silicon microring by integrating asymmetric Mie scatterers, which steer the system toward an EP [34], as well as through the use of external nano cylinder scatterers [35].

The interest in manipulating light transcends propagation controllability to topological phenomena at optical interfaces [36] and extends to surface plasmon polaritons (SPPs), which are evanescent electromagnetic waves coupled to charge oscillation at the metal–dielectric interfaces [37]. SPPs hold the promise of transcending the diffraction limit and enhancing light–matter interactions [38] and are envisioned to enable key applications in sensing [39,40,41], nanolasers [42,43,44], microscopy [45], spectroscopy [46,47], data transmission [48], and integrated optical communication [49,50]. Therefore, considerable efforts have been devoted to achieving robust and controllable manipulation of SPPs. Although conventional techniques such as nanoslits [51], catenary apertures [52], nanoantennas [53,54,55,56,57], and metasurfaces [58,59,60] have been explored to facilitate the robust and directional control of SPPs, these methods often suffer from considerable scattering losses, low extinction ratios, and complex manufacturing processes. Alternatively, non-Hermitian PT-symmetric photonic systems have recently emerged as a compelling alternative for manipulating SPPs at EPs. For instance, recent theoretical studies have shown that the modulation of PT-symmetric refractive index in a dielectric–metallic waveguide can enable unidirectional clocking for SPPs at EP [61] and the adjustment of Fermi energy and inter-ribbon distances in double-layer graphene nanoribbons to achieve unidirectional reflectionless SPP excitation in the mid-infrared range [62]. Moreover, metagratings have shown the potential for the unidirectional excitation of SPPs at EPs [63] and have been employed to achieve unidirectional SPP excitation at EPs in infrared [64], offering a novel and flexible approach for SPP manipulation.

Despite these advances, the potential for unidirectional SPP excitation at visible frequencies using non-Hermitian PT-symmetric metagratings remains unexplored. Such an approach could leverage symmetric complex optical potentials without relying on metallic materials in the metagrating design, simplifying the fabrication and integration into practical applications. In this work, we formulate a rigorous TCMT description of non-Hermitian metagratings that maps the complex permittivity modulation onto an effective non-Hermitian two-mode Hamiltonian, allowing us to analytically identify the exceptional points and their associated unidirectional SPP eigenstates (see Appendix A). Building on this framework, we demonstrate the unidirectional excitation of SPPs in the visible spectrum through permittivity modulation using a metal-free non-Hermitian metagrating composed of alternating Si–Ge nanostrips. This design benefits from a simpler fabrication process and leverages passive PT-symmetry, enabling the robust and energy-efficient control of SPPs in the visible spectrum, with potential applications in integrated photonic circuits, visible-band optical communications, and on-chip optical routing.

## 2. Materials and Methods

### 2.1. Theoretical Model

In this study, we investigate the unidirectional excitation of surface plasmon polaritons (SPPs) in the visible spectrum at exceptional points (EPs) under passive PT-symmetry. This is achieved by meticulously engineering the spatial distribution of the permittivity profile at a dielectric–metal interface using a non-Hermitian metallic-free metagrating that is driven to operate at EPs at the optimised spatial distribution of both the real ($\varepsilon_{r}$) and imaginary ($\varepsilon_{i}$) parts of the permittivity.

**Figure 1:**
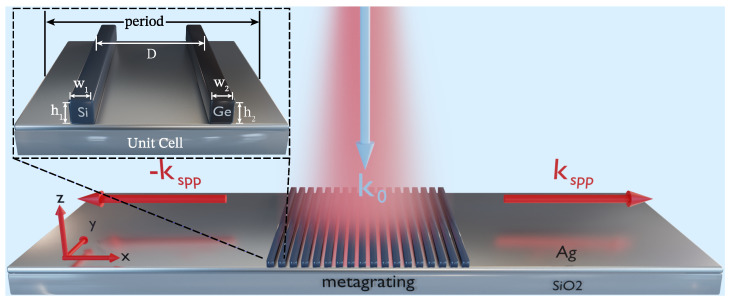
Schematic of the Si-Ge metagrating for the unidirectional excitation of SPP in the visible spectrum. The inset shows a unit cell of the metagrating with period Λ, comprising a Si nanostrip of width $w_{1}$ and height $h_{1}$ and a Ge nanostrip of width $w_{2}$ and height $h_{2}$, separated by a centre-to-centre distance *D*.

Figure 1 illustrates a schematic of the proposed structure, which consists of a silicon–germanium (Si-Ge) metagrating placed on top of a metallic (silver) and dielectric (air) interface. This configuration introduces a non-uniform spatial distribution of the dielectric refractive index along the silver–air boundary. The perturbation of the permittivity profile ϵ(x) at the metal–dielectric interface, in the presence of the metagrating, is denoted as Δϵ(x) and can be expressed under the assumption of a continuous passive PT-symmetric profile as follows [33,65]:

(1) $$\Delta \epsilon \left(x\right)=\Delta \epsilon \left[\cos\left(k_{\mathrm{spp}}x\right)-i\delta \sin\left(k_{\mathrm{spp}}x-\phi \right)\right].$$

Δϵ is the perturbation strength, δ, ϕ, and $k_{\mathrm{spp}}$ are the additional imaginary modulation, the relative phase shift between the real and imaginary parts of the permittivity perturbation, and the wavenumber of the excited SPP wave. Equation (1) can be further simplified as follows:

(2) $$\Delta \epsilon \left(x\right)=\Delta \epsilon_{r}e^{ik_{\mathrm{spp}}x}+\Delta \epsilon_{i}e^{-ik_{\mathrm{spp}}x},$$

where $\Delta \epsilon_{r}=\frac{\Delta \epsilon }{2}\left(1-\delta e^{-i\phi }\right)$, and $\Delta \epsilon_{i}=\frac{\Delta \epsilon }{2}\left(1+\delta e^{i\phi }\right)$. Consequently, the overall permittivity profile at the interface will be

(3) $$\epsilon \left(x\right)=\epsilon_{\mathrm{air}}+\Delta \epsilon_{r}e^{ik_{\mathrm{spp}}x}+\Delta \epsilon_{i}e^{-ik_{\mathrm{spp}}x}.$$

Physically, the Δϵ(x) term represents a complex periodic perturbation to the permittivity continuum of the Ag–Air interface. To realise the passive PT-symmetry condition $\varepsilon \left(x\right)\;=\;\varepsilon^{*}\left(-x\right)$, this spatial modulation is designed such that the real permittivity component (dominated by Si) forms an even function, while the imaginary component (dominated by Ge) forms an odd function. Referring to the formal equivalence between the Schrödinger equation in quantum mechanics and the paraxial wave equation in optics, Equation (1) indicates that the PT-symmetry condition for this optical potential, i.e., $\varepsilon \left(x\right)=\varepsilon^{*}\left(-x\right)$ [66], is satisfied when ϕ=0 or π.

It is well-known that the interaction between the free space electromagnetic field and metal surface charges increases the SPP momentum compared to free space, resulting in a momentum mismatch between light and SPPs. This mismatch can be addressed using various configurations, such as Kretschmann and Otto prism configurations or grating couplers, which align the momentum of light with that of the SPPs according to the SPP dispersion relation [37,67].

(4) $$k_{\mathrm{spp}}=k_{0}\frac{\epsilon_{d}\epsilon_{m}}{\epsilon_{d}+\epsilon_{m}}$$

Herein, $\epsilon_{d}$ and $\epsilon_{m}$ denote the permittivities of the dielectric, the thin metal film, $k_{0}$, represents the wave numbers of the incident wave in free space, and $k_{\mathrm{spp}}$ is the wavenumber of the SPP excited at the interface.

Hence, to excite SPPs along the air–silver interface with normally incident light on the metagrating, the metagrating’s period Λ should be calibrated to address the momentum mismatch with the incident light to enable efficient coupling from the diffracted electromagnetic wave to the SPP evanescent wave propagating at the interface, and Λ can be mathematically deduced from the SPP dispersion relation, where $k_{0}$ is given by $k_{0}=\frac{2\pi }{\lambda_{0}}$, with $\lambda_{0}$ being the wavelength of the normally incident wave on the metagrating and $\frac{2\pi }{\Lambda }=Re\left(k_{\mathrm{spp}}\right)$. The coupling mechanism can be precisely controlled through the geometric and material parameters of the metagrating.

**Figure 2:**
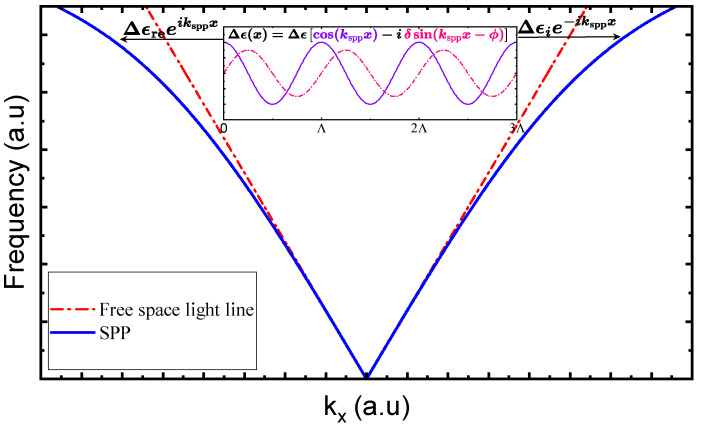
Dispersion curve for free space light and SPP illustrating the coupling mechanism and the excitation of surface plasmon polaritons (SPPs) along the air–silver interface using a metagrating. The periodic modulation of the permittivity enables phase matching and efficient coupling of normally incident light to SPPs. The perturbation, shown as $\Delta \epsilon \left(x\right)=\Delta \epsilon [\cos\left(k_{\mathrm{spp}}x\right)-i\delta \sin\left(k_{\mathrm{spp}}x-\phi \right)]$, allows control of the coupling direction by adjusting the metagrating parameters.

In the absence of the metagrating, the permittivity modulation Δε in Equation (1) vanishes; so, the normally incident light cannot couple to the SPP mode, and no SPPs are excited. However, when the metagrating is introduced with the calibrated periodicity, the periodic modulation of the permittivity, as expressed in Equation (2), provides the phase-matching necessary to excite SPPs. By adjusting this perturbation, which is determined by the metagrating parameters, the coupling to the right and left of the metagrating can be effectively controlled. Additionally, unidirectional excitation can be achieved by manipulating the parameters to derive the passive PT-symmetric system to operate at an EP, as illustrated in Figure 2.

The impact of this continuous perturbing modulation of the dielectric permittivity at the interface on the excitation of the SPP evanescent wave is quantified using the TCMT framework detailed in Appendix A, which maps the complex permittivity modulation onto an effective non-Hermitian two-mode Hamiltonian for the right- and left-propagating SPPs. Within this description, the steady-state solution of the coupled-mode equations shows that the field magnitude ratio of the right ($H_{+}$) and left ($H_{-}$) branches of the supported transverse magnetic (TM) mode at the interface is linearly proportional to the ratio of the first-order Fourier coefficients of the complex permittivity modulation associated with the two propagation directions,

(5) $$\left|\frac{H_{+}}{H_{-}}\right|=\left|\frac{\Delta \epsilon_{i}}{\Delta \epsilon_{r}}\right|.$$

From Equations (1), (4) and (5), we discern that the asymmetric ratio $\left|\frac{\Delta \epsilon_{i}}{\Delta \epsilon_{r}}\right|$ in the introduced permittivity modulation underpins the momentum compensation for SPP propagation towards the right or left direction on the surface. Hereby, it is possible to finely control this directional propagation by controlling the strength of the permittivity perturbation. Although implementing a continuous permittivity perturbation is challenging in practical scenarios, it can be effectively approximated through discrete spatial modulation using metagrating nanostrips, and the perturbation strength can then be controlled by varying the geometric parameters of these nanostrips ($w_{1}$, $h_{1}$, $w_{2}$, $h_{2}$), along with their centre-to-centre separation distance *D* as shown in Figure 1. In practice, the effective modulation depth Δε and the relative imaginary modulation parameter δ are determined by the complex permittivities of Si and Ge at $\lambda_{0}=633\;\mathrm{nm}$ and by the filling factors of the two materials within a unit cell (set by $w_{1}$, $w_{2}$, $h_{1}$, and $h_{2}$). The phase ϕ arises from the relative shift of the strips inside the period Λ, which controls the relative phase of the forward and backward Bloch components and, thus, the balance between the right and left SPP channels.

Consequently, the momentum coupling from the incident free-space light to either the left or right branch of the excited SPP can be adjusted, as visually depicted in the dispersion curve in Figure 2. Within the TCMT framework (Appendix A), the complex eigenvalues associated with the two counter-propagating SPP modes are

(6) $$\lambda_{1,2}\approx 1\pm \sqrt{\frac{\Delta \epsilon_{i}\Delta \epsilon_{r}}{2}}\approx 1\pm \frac{\Delta \epsilon }{2\sqrt{2}}\sqrt{1-\delta^{2}+2i\delta \sin\phi }.$$

The eigenvalues coalesce when the square-root term vanishes, i.e., when $\Delta \epsilon_{r}\Delta \epsilon_{i}=0$. Physically, this coalescence arises from the interplay between the coherent coupling rate (*J*) and the effective gain/loss contrast (Γ), as defined in the temporal coupled-mode theory description in Appendix A (see Equation (A6)). When these two competing rates are perfectly balanced (|J|=|Γ|), the system undergoes a phase transition where the two counter-propagating modes can no longer exist as distinct states, causing them to collapse into a single unidirectional eigenstate. In that case, one of the directional coupling channels is suppressed, and the two eigenmodes collapse into a single unidirectional SPP state, aligned with either $H_{+}$ or $H_{-}$ (see Equation (5)). Using the explicit expressions for $\Delta \epsilon_{r}$ and $\Delta \epsilon_{i}$, this degeneracy occurs at δ=±1 and ϕ=0 or π, where the system sits at an exceptional point with a real eigenvalue spectrum.

**Figure 3:**
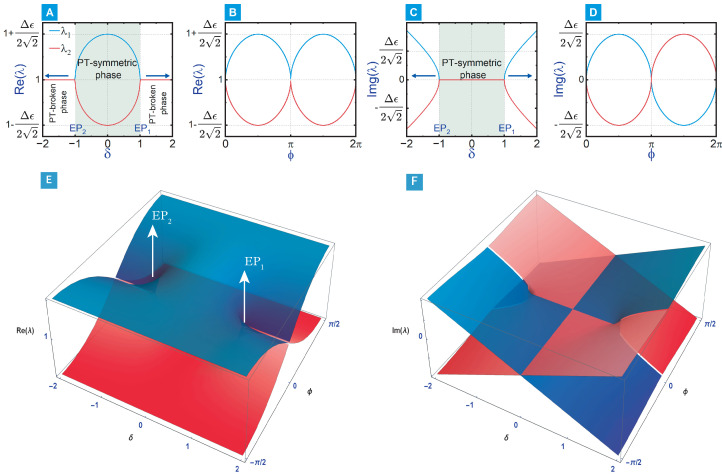
The evolution of the eigenvalues in 1D and 2D parameter space of the non-Hermitian system. (**A**) The eigenvalue real part and (**C**) imaginary part with varying δ when ϕ=0. (**B**) The eigenvalue real part and (**D**) imaginary part with varying ϕ when δ=1. (**E**) Real and (**F**) imaginary parts of the system eigenvalues $\lambda_{1}$ (blue) and $\lambda_{2}$ (red) in the 2D parameter space, when δ is varied from −2 to 2, and ϕ is varied from $-\frac{\pi }{2}$ to $\frac{\pi }{2}$. The bifurcation occurs at (δ,ϕ)=(±1,0), corresponding to the EPs of the system.

The non-Hermitian system eigenvalue evolution in the 1D and 2D parameter spaces is shown in panels A–D and E–F of Figure 3, respectively. A bifurcation occurs when the square-root term in Equation (6), $1-\delta^{2}+2i\delta \sin\phi$, vanishes, marking the topological transition from the PT-symmetric to the PT-broken phase, as seen in Figure 3A,C. This condition is satisfied at (δ,ϕ)=(±1,0) and (±1,π), which, in terms of the effective modulation coefficients, correspond to $\Delta \epsilon_{r}=0$ or $\Delta \epsilon_{i}=0$, i.e., the two EPs of the system. As illustrated by the Riemann surfaces in Figure 3E,F, these four points organise into two symmetry-related pairs: the transformation (δ,ϕ)→(−δ,ϕ+π) leaves $\Delta \epsilon_{r}$ and $\Delta \epsilon_{i}$ invariant, so that (δ,ϕ)=(1,0) is equivalent to (−1,π), and (−1,0) is equivalent to (1,π). Consequently, the parameter space hosts two physically distinct EPs that appear symmetrically about δ=0.

### 2.2. Numerical Simulation

We have performed a full-wave modelling for the structure depicted in Figure 1 using COMSOL Multiphysics commercial software (version: Comsol 6.0) to verify the theoretical model. In the simulation, a Gaussian beam profile was implemented as the source with a waist radius covering the metagrating. The incident light was set at the visible wavelength of $\lambda_{0}=633$ nm, with the polarisation oriented along the x-direction, the scattering boundary condition at the top, and perfectly matched layers (PML) of λ thickness at the left, right, and bottom, and the domain was meshed using the physics-controlled `extremely fine’ setting, with a maximum element size of λ/60 and λ/30 localised at the nanostrips and the grating Ag-Air interface, respectively. The metagrating period Λ was set at 616 nm to meet the phase matching condition for SPP excitation as per the dispersion relation in Equation (4). After that, the metagrating nanostrips’ geometric parameters, their separation distance, and their position along the grating unit cell are carefully optimised to approximate the theoretical continuous PT-symmetric modulated permittivity profile in Equation (2) to further control the coupling strengths of the excited SPPs to the left and right directions of the metagrating opting to maximise or minimise the ratio $\left|\frac{\Delta \epsilon_{i}}{\Delta \epsilon_{r}}\right|$, where the system EPs occur, and the SPP modes coalesce, as inferred from Equations (5) and (6).

In a unit cell of the metagrating, the nanostrips are made of silicon (Si), which has nearly real permittivity at the operating wavelength of 633 nm [68]; hence, it contributes to the real modulation of the permittivity. On the other hand, the second nanostrip is made of germanium (Ge), which has relatively high real and imaginary permittivity at the chosen wavelength [69] and contributes to both the real and imaginary modulation of the permittivity profile at the interface. The permittivity of the underlying Ag film was taken from Johnson and Christy experimental data [70]. Here, we note that while the inclusion of absorptive Ge nanostrips introduces ohmic losses, these are strictly localised to the metagrating region. Consequently, these losses primarily affect the coupling efficiency (the ratio of incident photon flux to launched SPP power) but do not degrade the propagation length or confinement of the excited SPP. Once launched, the SPP propagates along the bare Ag–Air interface, where its decay characteristics are governed solely by the intrinsic losses of the silver film at the operating wavelength.

## 3. Results and Discussion

**Figure 4:**
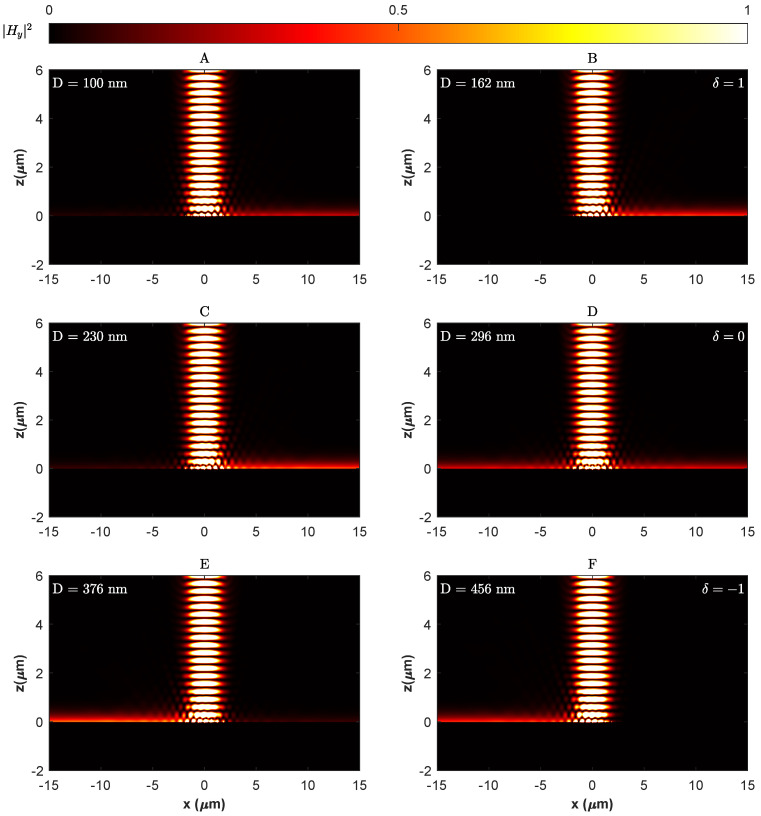
Intensity of the transverse magnetic field $|H_{y}{|}^{2}$ of the SPP TM mode excited by a metagrating consisting of seven-unit cells for different nanostrip separation distances: (**A**) D=100 nm, (**B**) D=162 nm, (**C**) D=230 nm, (**D**) D=296 nm, (**E**) D=376 nm, (**F**) D=456 nm.

The intensity of the transverse magnetic field $|H_{y}{|}^{2}$ of the SPP TM mode excited by a metagrating consisting of seven-unit cells in total with alternating Si-Ge nanostrips is plotted in Figure 4. At the optimised geometric parameters of $w_{1}=36$ nm and $h_{1}=30$ nm for the Si nanostrip, $w_{2}=64$ nm and $h_{2}=30$ nm for the Ge nanostrip and nanostrips, and a centre-to-centre separation distance D=162 nm, a unidirectional excitation of SPP can be observed along the +x direction to the right of the metagrating as shown in Figure 4B. Hence, a separation distance equivalent to approximately a quarter grating period between the Si-Ge nanostrips in a unit cell of the metagrating at their optimised geometric parameters is required to tune the imaginary modulation of the metagrating δ to a value of 1 necessary to break the PT-symmetry and drive the system into the first exceptional point at which a unidirectional excitation of SPP to the right direction occurs.

**Figure 5:**
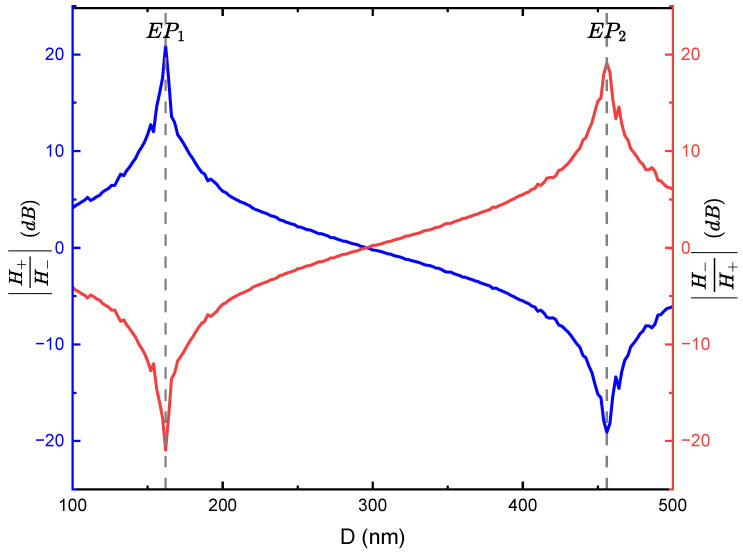
Field extinction ratio of the right SPP mode to left SPP mode (blue) and the left SPP mode to right SPP mode (red) versus nanostrips’ centre-to-centre separation distance *D* when the geometric parameters are optimised at $w_{1}=36$ nm, $h_{1}=30$ nm, $w_{2}=64$ nm, and $h_{2}=30$ nm, respectively.

As shown in Figure 5, the field contrast of the left and right SPP modes at the optimised geometric parameters is controlled by adjusting the separation distance *D* between the nanostrips in the grating unit cell. This adjustment controls the strength of the imaginary modulation δ of the permittivity perturbation, thereby altering the ratio $\left|\frac{\Delta \epsilon_{i}}{\Delta \epsilon_{r}}\right|$ (refer to Equation (3)) and facilitating the control of SPP excitation (refer to Equation (5)). As illustrated in Figure 4A, at a separation distance of 100 nm, the field contrast $\left|\frac{H_{+}}{H_{-}}\right|$ was ≈4.11 dB. However, it increased dramatically to ≈20.9 dB at a separation distance of 162 nm (Figure 4B) corresponding to δ≈1 and the first exceptional point of the non-Hermitian metagrating. Notably, Figure 5 demonstrates that a high field contrast (>10 dB) is maintained over a separation range of approximately D=162±10 nm, indicating that the unidirectional excitation is robust against typical fabrication tolerances in electron beam lithography.

As the separation distance further increased beyond 162 nm, the imaginary modulation index δ became less than 1, which is physically translated to more light being coupled to the left SPP mode, but it still couples more efficiently to the right SPP mode, and the field contrast decreased accordingly.

**Figure 6:**
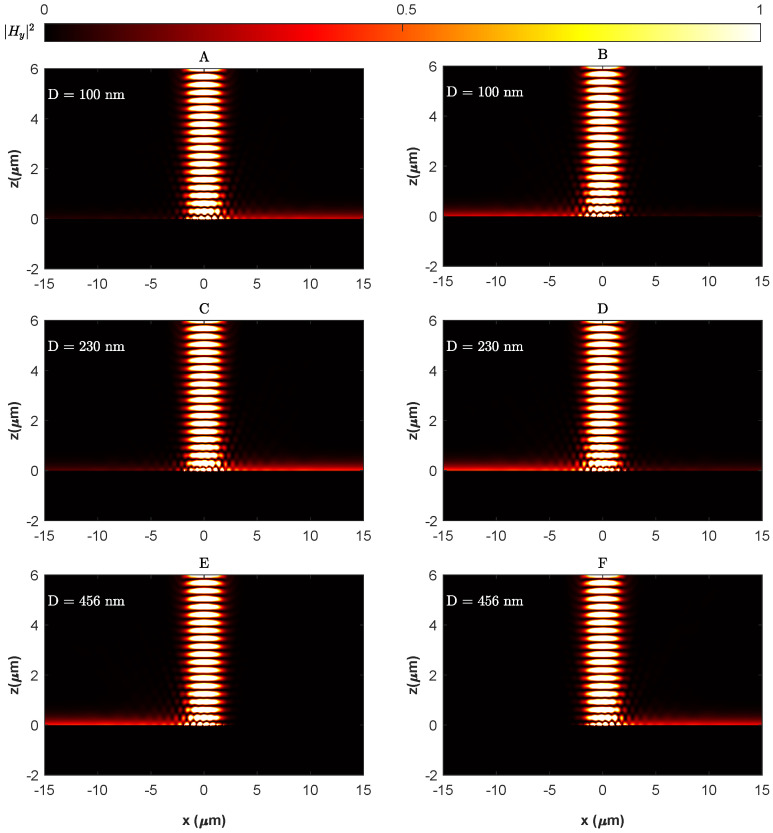
Intensity of the transverse magnetic field $|H_{y}{|}^{2}$ of the excited SPP when the metagrating unit cells are composed of Si-Ge nanostrips (panels (**A**,**C**,**E**)) and when the nanostrips positions are swapped to form unit cells of Ge-Si (panels (**B**,**D**,**F**)) showing complete reversal of the SPP excitation patterns, indicating a phase shift of ϕ=π in the permittivity modulation described in Equation (1). The first scenario corresponds to a system with EPs at (δ,ϕ)=(±1,0) and the second with EPs at (δ,ϕ)=(∓1,π).

Remarkably, the imaginary modulation δ reaches approximately 0, at which the field contrast is ≈0 dB when the separation distance reaches a value of D=296 nm. In this case, the effective dielectric constant ϵ(x) is real, and the permittivity profile is effectively Hermitian in agreement with what is expected from scattering of light from subwavelength apertures [71] and as inferred from Equations (2) and (5) when $\Delta \epsilon_{i}=\Delta \epsilon_{r}$; this is evident by the symmetrically equal excitation of SPP to the left and right of the metagrating as seen in Figure 4D. Beyond this point, the light tends to couple more efficiently to the left SPP mode, and the field contrast ratio plummeted dramatically to ≈19.1 dB at D=456 nm, corresponding to δ=−1 and the second exceptional point of the system, with SPP steering towards unidirectional excitation in the −x direction as presented in Figure 4F. The previous results demonstrate that the permittivity profile of the Si-Ge metagrating conforms to a passive PT-symmetric model, as delineated in Equation (1), that expressed the introduced permittivity perturbation at the interface as a periodic perturbing variation in the real and imaginary parts of the permittivity, expanded in terms of Fourier series of backward and forward Bloch modes. The induced phase shift ϕ in the model can be adjusted while preserving the PT-symmetry by altering the positioning of the nanostrips within the unit cells of the metagrating. The effect of swapping the positions of the Si and Ge nanostrips on the SPP excitation is elucidated in Figure 6, which shows that exchanging the Si and Ge positions in the unit cell yielded a complete reversal of the SPP pattern, confirming an induced phase shift of ϕ=π in line with the mirror symmetry in the parameter space as seen in Figure 3.

Figure 5 elaborates the dependence of SPP excitation and the right and left SPP modes extinction ratio on the nanostrips’ separation distance, where, at EPs, a field amplitude extinction ratio ≈20 dB equivalent to ≈40 dB optical power extinction ratio can be achieved in the visible frequencies, outperforming those achieved in conventional planar structures and apertures, as summarised in Table 1.

While conventional couplers rely on precise interference effects accumulated over a specific interaction length—where the extinction ratio is essentially limited by phase matching constraints—the proposed EP-based device leverages a topological singularity. At the EP, the eigenstates coalesce, leading to the mathematical collapse of the backward mode. This renders the unidirectionality an intrinsic property of the system’s eigenstates rather than a result of distributed interference, fundamentally enabling superior extinction ratios.

## 4. Conclusions

In this study, we successfully demonstrated a robust unidirectional excitation of SPPs at EPs in the visible spectrum with an intensity extinction ratio of about 40 dB that outperforms those achieved in conventional planar structures and apertures. This was solely achieved by engineering the parameters of a non-metallic Si-Ge metagrating designed to approximate a passive PT-symmetric optical potential, optimised to operate at EPs. These findings highlight the potential of non-Hermitian metagratings for advanced applications in optical integrated circuits, optical routing, and communication systems, where robust and flexible control of light at the nanoscale is crucial.

## Figures and Tables

**Table 1:** Comparison of unidirectional SPP excitation techniques in the visible/near-visible range.

Technique	Mechanism	Extinction Ratio (dB)	Footprint	Ref.
Si-Ge Metagrating	EP Coalescence	≈40 (Sim)	∼4.3 μm	This Work
Asymmetric Nanoslit	FP Cavity Interference	∼16 (Sim)	∼0.4 μm	[51]
Catenary Apertures	Geometric Phase	∼27 (Sim)	∼0.6 μm	[52]
Crossed Nanoantennas	Dipole Interference	∼17.6 (Sim)	∼1.0 μm	[57]
Plasmonic Apertures	Polarisation Interference	∼14 (Exp)	Multi-column	[60]
Metasurface	Phase Discontinuity	∼14 (Sim)	∼17 μm	[59]

## Data Availability

The original contributions presented in this study are included in the article. Further inquiries can be directed to the corresponding authors.

## Abbreviations

The following abbreviations are used in this manuscript:

DP	Diabolic point
EP	Exceptional point
Ge	Germanium
PML	Perfectly matched layers
PT	Parity–time
SPP	Surface plasmon polariton
Si	Silicon
Si-Ge	Silicon–germanium
TCMT	Temporal coupled mode theory
TM	Transverse magnetic

## Appendix A. Temporal Coupled-Mode Description and Exceptional Points Derivation

In this Appendix, we present an effective temporal coupled-mode description of the Si–Ge metagrating and derive the conditions for unidirectional SPP excitation and the emergence of exceptional points. Rather than solving Maxwell’s equations explicitly in the presence of the grating, we treat the two counter-propagating SPPs at the air–silver interface as a pair of coupled non-Hermitian modes, constrained by reciprocity and passive PT symmetry. This provides a compact and physical route to Equations (5) and (6) in the main text.

### Appendix A.1. Effective Two-Mode Model

We consider the SPPs supported at the air–silver interface without the metagrating as a pair of degenerate counter-propagating modes with complex frequency

(A1) $${\tilde{\omega }}_{0}=\omega_{0}-i\gamma_{0},$$

where $\omega_{0}$ is the SPP resonance frequency, and $\gamma_{0}$ accounts for the intrinsic absorption and radiation loss. Introducing the metagrating breaks the continuous translational symmetry and creates a periodic complex permittivity modulation at the interface. In the main text, this modulation is written as

(A2) $$\Delta \epsilon \left(x\right)=\Delta \epsilon \left[\cos\left(k_{\mathrm{spp}}x\right)-i\delta \sin\left(k_{\mathrm{spp}}x-\phi \right)\right],$$

whose dominant spatial Fourier components at $\pm k_{\mathrm{spp}}$ can be expressed as

(A3) $$\Delta \epsilon \left(x\right)=\Delta \epsilon_{r}e^{ik_{\mathrm{spp}}x}+\Delta \epsilon_{i}e^{-ik_{\mathrm{spp}}x}$$

with $\Delta \epsilon_{r}$ and $\Delta \epsilon_{i}$ given in terms of the modulation parameters by

(A4) $$\Delta \epsilon_{r}=\frac{\Delta \epsilon }{2}\left(1-\delta e^{-i\phi }\right)\;\Delta \epsilon_{i}=\frac{\Delta \epsilon }{2}\left(1+\delta e^{i\phi }\right).$$

Physically, the complex coefficients $\Delta \epsilon_{r}$ and $\Delta \epsilon_{i}$ control the scattering of normally incident light into the right- and left-going SPP channels, respectively.

We denote by $a_{+}\left(t\right)$ and $a_{-}\left(t\right)$ the slowly varying amplitudes of the right- and left-propagating SPPs at the interface, normalised such that $|a_{\pm }{|}^{2}$ is proportional to the SPP power. In the absence of external excitation, their temporal evolution can be written in vector form as

(A5) $$\frac{d}{dt}\left(\begin{array}{c} a_{+}\\ a_{-} \end{array}\right)=-iH_{\mathrm{eff}}\left(\begin{array}{c} a_{+}\\ a_{-} \end{array}\right),$$

where $H_{\mathrm{eff}}$ is a 2×2 effective non-Hermitian Hamiltonian. For a passive reciprocal structure with a complex refractive-index modulation, $H_{\mathrm{eff}}$ must be symmetric and satisfy a PT-like constraint. A convenient parametrisation is

(A6) $$H_{\mathrm{eff}}=\left(\begin{array}{cc} {\tilde{\omega }}_{0}+i\Gamma & J\\ J & {\tilde{\omega }}_{0}-i\Gamma \end{array}\right),$$

where J∈R describes coherent coupling between the two SPP channels (arising primarily from the real part of the modulation), and Γ∈R represents an effective gain/loss contrast between them (arising from the imaginary part of the modulation). Physically, these effective Hamiltonian parameters are determined by the Fourier coefficients of the permittivity perturbation, $\Delta \epsilon_{r}$ and $\Delta \epsilon_{i}$ (defined in Equation (A4)). As detailed in the main text, these coefficients are engineered parameters: they are directly derived from the permittivity perturbation at the interface, which is controlled by the interplay between the complex permittivities of the Si and Ge nanostrips and their geometric filling factors within the unit cell. The non-Hermitian behavior arises when these geometric and material parameters are tuned such that the resulting Fourier components determine the critical parameters δ and ϕ to satisfy the EP condition $1-\delta^{2}+2i\delta \sin\phi =0$ (derived in Equation (A26)). The matrix in Equation (A6) is invariant under the combined action of parity (exchange $a_{+}↔a_{-}$) and time reversal (complex conjugation and t→−t), i.e., it realises a passive PT-symmetric dimer.

The eigenfrequencies of Equation (A6) are

(A7) $$\omega_{\pm }={\tilde{\omega }}_{0}\pm \sqrt{J^{2}-\Gamma^{2}},$$

revealing the characteristic square-root branch structure of non-Hermitian two-mode systems. In the PT-symmetric phase |J|>|Γ|, the splitting is real, whereas in the PT-broken phase |J|<|Γ|, the two modes form a complex conjugate pair. The exceptional points (EPs) occur when the radicand vanishes,

(A8) $$J^{2}=\Gamma^{2},$$

at which both the eigenvalues and eigenvectors of $H_{\mathrm{eff}}$ coalesce, and the matrix becomes defective.

### Appendix A.2. External Coupling and SPP Directionality

We now include excitation by a normally incident TM-polarised plane wave with complex amplitude $s_{\mathrm{in}}$ impinging on the metagrating from above. Within temporal coupled-mode theory, the driven steady-state dynamics at frequency ω can be expressed as

(A9) $$\left(\omega I-H_{\mathrm{eff}}\right)\left(\begin{array}{c} a_{+}\\ a_{-} \end{array}\right)=K\;s_{\mathrm{in}},$$

where I is the identity matrix, and K is a two-component coupling vector that encodes how the input plane wave feeds each SPP channel.

To obtain K in a rigorous way, we use perturbative coupling theory. The coupling strength of a normally incident plane wave (with transverse momentum $k_{\mathrm{inc},x}\approx 0$) to a specific SPP mode is proportional to the overlap integral over one period Λ,

(A10) $$\kappa \propto \int_{0}^{\Lambda }{\vec{E}}_{\mathrm{mode}}^{*}\left(x\right)\;\Delta \epsilon \left(x\right)\;{\vec{E}}_{\mathrm{inc}}\left(x\right)\;dx,$$

where we absorb the slowly varying incident field into the overall proportionality constant.

We choose the right-propagating SPP mode as $E_{+}\left(x\right)\propto e^{-ik_{\mathrm{spp}}x}$, so that $E_{+}^{*}\left(x\right)\propto e^{+ik_{\mathrm{spp}}x}$. Substituting Equation (A3) into Equation (A10) gives

(A11) $$\begin{array}{cc} \kappa_{+} & \propto \int_{0}^{\Lambda }e^{+ik_{\mathrm{spp}}x}\left(\Delta \epsilon_{r}e^{ik_{\mathrm{spp}}x}+\Delta \epsilon_{i}e^{-ik_{\mathrm{spp}}x}\right)dx\\ \; & \propto \int_{0}^{\Lambda }\left(\Delta \epsilon_{r}e^{2ik_{\mathrm{spp}}x}+\Delta \epsilon_{i}\right)dx. \end{array}$$

The rapidly oscillating term $e^{2ik_{\mathrm{spp}}x}$ averages to zero over one period; so, only the constant term contributes. Hence,

(A12) $$\kappa_{+}=\eta \;\Delta \epsilon_{i},$$

where η is a real constant encoding the mode normalisation and vertical field overlap.

Similarly, the left-propagating mode is taken as $E_{-}\left(x\right)\propto e^{+ik_{\mathrm{spp}}x}$, so that $E_{-}^{*}\left(x\right)\propto e^{-ik_{\mathrm{spp}}x}$. The corresponding overlap integral yields

(A13) $$\begin{array}{cc} \kappa_{-} & \propto \int_{0}^{\Lambda }e^{-ik_{\mathrm{spp}}x}\left(\Delta \epsilon_{r}e^{ik_{\mathrm{spp}}x}+\Delta \epsilon_{i}e^{-ik_{\mathrm{spp}}x}\right)dx\\ \; & \propto \int_{0}^{\Lambda }\left(\Delta \epsilon_{r}+\Delta \epsilon_{i}e^{-2ik_{\mathrm{spp}}x}\right)dx, \end{array}$$

so that the oscillatory term again vanishes, and we obtain

(A14) $$\kappa_{-}=\eta \;\Delta \epsilon_{r}.$$

Equations (A12) and (A14) show that, under normal incidence, the incident light couples to the right-going SPP via the $-k_{\mathrm{spp}}$ Fourier component $\Delta \epsilon_{i}$ and to the left-going SPP via the $+k_{\mathrm{spp}}$ component $\Delta \epsilon_{r}$, consistent with the momentum-matching picture.

Solving Equation (A9) for the steady-state amplitudes gives

(A15) $$\left(\begin{array}{c} a_{+}\\ a_{-} \end{array}\right)={\left(\omega I-H_{\mathrm{eff}}\right)}^{-1}K\;s_{\mathrm{in}}.$$

Close to the SPP resonance ($\omega \approx \omega_{0}$) and for weak modulation (so that *J* and Γ are small compared to $\omega_{0}$), the denominator affecting both $a_{+}$ and $a_{-}$ is nearly the same. As a result, the ratio of the excited SPP amplitudes is, to a very good approximation, determined solely by the ratio of the coupling coefficients:

(A16) $$\frac{a_{+}}{a_{-}}≃\frac{\kappa_{+}}{\kappa_{-}}=\frac{\Delta \epsilon_{i}}{\Delta \epsilon_{r}}.$$

Identifying $H_{\pm }$ with the magnetic field amplitudes of the right- and left-propagating SPPs leads directly to the expression used in the main text,

(A17) $$\left|\frac{H_{+}}{H_{-}}\right|=\left|\frac{\Delta \epsilon_{i}}{\Delta \epsilon_{r}}\right|=\left|\frac{1+\delta e^{i\phi }}{1-\delta e^{-i\phi }}\right|,$$

which quantifies the SPP directionality in terms of the complex permittivity modulation.

### Appendix A.3. Exceptional Points and Unidirectional Excitation

The EPs of the non-Hermitian SPP dimer are governed by Equation (A8). Physically, *J* is induced predominantly by the conservative (real) part of the permittivity modulation, while Γ arises from the dissipative (imaginary) part. For weak modulation, it is sufficient to assume that both *J* and Γ are linear in the Fourier amplitudes $\Delta \epsilon_{r}$ and $\Delta \epsilon_{i}$ introduced in Equation (A3). To leading order, we may therefore write

(A18) $$J=j_{r}\;\Delta \epsilon_{r}+j_{i}\;\Delta \epsilon_{i}+O\left(\Delta \epsilon^{2}\right),\;\Gamma =g_{r}\;\Delta \epsilon_{r}+g_{i}\;\Delta \epsilon_{i}+O\left(\Delta \epsilon^{2}\right),$$

where $j_{r,i}$ and $g_{r,i}$ are real constants fixed by the geometry and the passive PT symmetry of the structure. Substituting into $J^{2}-\Gamma^{2}$ and retaining only the leading (quadratic) terms in $\Delta \epsilon_{r,i}$ gives

(A19) $$\begin{array}{cc} J^{2}-\Gamma^{2} & ≃\left(j_{r}^{2}-g_{r}^{2}\right)\;\Delta \epsilon_{r}^{2}+\left(j_{i}^{2}-g_{i}^{2}\right)\;\Delta \epsilon_{i}^{2}+2\left(j_{r}j_{i}-g_{r}g_{i}\right)\;\Delta \epsilon_{r}\Delta \epsilon_{i}. \end{array}$$

From the microscopic Maxwell description, we know that the EP manifold includes the two limiting cases $\Delta \epsilon_{r}=0$ with $\Delta \epsilon_{i}\neq 0$ and $\Delta \epsilon_{i}=0$ with $\Delta \epsilon_{r}\neq 0$, i.e., $J^{2}-\Gamma^{2}$ must vanish along both coordinate axes in the $\left(\Delta \epsilon_{r},\Delta \epsilon_{i}\right)$ plane. This requires the coefficients of $\Delta \epsilon_{r}^{2}$ and $\Delta \epsilon_{i}^{2}$ in Equation (A19) to vanish,

(A20) $$j_{r}^{2}=g_{r}^{2},\;j_{i}^{2}=g_{i}^{2},$$

so that the leading dependence of $J^{2}-\Gamma^{2}$ reduces to the mixed term

(A21) $$J^{2}-\Gamma^{2}\propto \Delta \epsilon_{r}\Delta \epsilon_{i},$$

which is the unique quadratic form consistent with the known EP conditions of the underlying Maxwell problem.

Using Equation (A4), the product of the two Fourier coefficients is

(A22) $$\begin{array}{cc} \Delta \epsilon_{r}\Delta \epsilon_{i} & =\frac{\Delta \epsilon^{2}}{4}\left[1-\delta^{2}+\delta \left(e^{i\phi }-e^{-i\phi }\right)\right]\\ \; & =\frac{\Delta \epsilon^{2}}{4}\left(1-\delta^{2}+2i\delta \sin\phi \right). \end{array}$$

Within the TCMT picture, the eigenfrequencies in Equation (A7) can therefore be written as

(A23) $$\omega_{\pm }={\tilde{\omega }}_{0}\pm C\sqrt{\Delta \epsilon_{r}\Delta \epsilon_{i}},$$

where *C* is a real proportionality constant that depends on the specific geometry and normalisation conventions. Substituting Equation (A22) into Equation (A23) gives

(A24) $$\omega_{\pm }={\tilde{\omega }}_{0}\pm C\;\frac{\Delta \epsilon }{2}\sqrt{1-\delta^{2}+2i\delta \sin\phi }.$$

It is convenient to introduce the dimensionless eigenvalues

(A25) $$\lambda_{1,2}\equiv \frac{\omega_{\pm }}{{\tilde{\omega }}_{0}}.$$

For weak modulation, Δϵ is small; so, the splitting $|\omega_{\pm }-{\tilde{\omega }}_{0}|$ is much smaller than $|{\tilde{\omega }}_{0}|$. Dividing Equation (A24) by ${\tilde{\omega }}_{0}$ and absorbing the geometry-dependent factor $C/{\tilde{\omega }}_{0}$ into the definition of the overall perturbation strength, we obtain

(A26) $$\lambda_{1,2}\approx 1\pm \frac{\Delta \epsilon^{\prime }}{2\sqrt{2}}\sqrt{1-\delta^{2}+2i\delta \sin\phi },$$

which coincides with Equation (6) in the main text.

The EPs correspond to the coalescence of these eigenvalues, which occurs when the square root in Equation (A26) vanishes:

(A27) $$1-\delta^{2}+2i\delta \sin\phi =0.$$

Separating the real and imaginary parts yields

(A28) $$1-\delta^{2}=0,\;\delta \sin\phi =0;$$

so, the EPs are located at

(A29) δ=±1,ϕ=0orπ.

At these parameter values either $\Delta \epsilon_{r}$ or $\Delta \epsilon_{i}$ vanishes; so Equation (A17) implies

(A30) $$\left|\frac{H_{+}}{H_{-}}\right|\to \infty \;\mathrm{or}\;\left|\frac{H_{+}}{H_{-}}\right|\to 0,$$

corresponding to strictly unidirectional SPP excitation to the right or to the left. The TCMT description thus provides a compact and physically transparent link between the location of exceptional points in the (δ,ϕ) parameter space and the observed unidirectional SPP transport in the Si–Ge metagrating.

## References

[1] Meng H., Ang Y.S., Lee C.H. (2024). Exceptional points in non-Hermitian systems: Applications and recent developments. Appl. Phys. Lett..

[2] Bender C.M., Boettcher S. (1998). Real spectra in non-Hermitian Hamiltonians having P T symmetry. Phys. Rev. Lett..

[3] Li A., Wei H., Cotrufo M., Chen W., Mann S., Ni X., Xu B., Chen J., Wang J., Fan S. (2023). Exceptional points and non-Hermitian photonics at the nanoscale. Nat. Nanotechnol..

[4] Teller E. (1937). The Crossing of Potential Surfaces. J. Phys. Chem..

[5] Heiss W. (2012). The physics of exceptional points. J. Phys. A Math. Theor..

[6] Makris K.G., El-Ganainy R., Christodoulides D., Musslimani Z.H. (2008). Beam dynamics in PT symmetric optical lattices. Phys. Rev. Lett..

[7] Klaiman S., Günther U., Moiseyev N. (2008). Visualization of branch points in PT-symmetric waveguides. Phys. Rev. Lett..

[8] El-Ganainy R., Makris K., Christodoulides D., Musslimani Z.H. (2007). Theory of coupled optical PT-symmetric structures. Opt. Lett..

[9] Longhi S. (2009). Bloch oscillations in complex crystals with PT symmetry. Phys. Rev. Lett..

[10] Rüter C.E., Makris K.G., El-Ganainy R., Christodoulides D.N., Segev M., Kip D. (2010). Observation of parity–time symmetry in optics. Nat. Phys..

[11] Guo A., Salamo G.J., Duchesne D., Morandotti R., Volatier-Ravat M., Aimez V., Siviloglou G.A., Christodoulides D.N. (2009). Observation of PT-symmetry breaking in complex optical potentials. Phys. Rev. Lett..

[12] Miri M.A., Alu A. (2019). Exceptional points in optics and photonics. Science.

[13] Chang L., Jiang X., Hua S., Yang C., Wen J., Jiang L., Li G., Wang G., Xiao M. (2014). Parity–time symmetry and variable optical isolation in active–passive-coupled microresonators. Nat. Photonics.

[14] Brandstetter M., Liertzer M., Deutsch C., Klang P., Schöberl J., Türeci H.E., Strasser G., Unterrainer K., Rotter S. (2014). Reversing the pump dependence of a laser at an exceptional point. Nat. Commun..

[15] Peng B., Özdemir Ş.K., Liertzer M., Chen W., Kramer J., Yılmaz H., Wiersig J., Rotter S., Yang L. (2016). Chiral modes and directional lasing at exceptional points. Proc. Natl. Acad. Sci. USA.

[16] Miao P., Zhang Z., Sun J., Walasik W., Longhi S., Litchinitser N.M., Feng L. (2016). Orbital angular momentum microlaser. Science.

[17] Feng L., Wong Z.J., Ma R.M., Wang Y., Zhang X. (2014). Single-mode laser by parity-time symmetry breaking. Science.

[18] Hodaei H., Miri M.A., Hassan A.U., Hayenga W.E., Heinrich M., Christodoulides D.N., Khajavikhan M. (2016). Single mode lasing in transversely multi-moded PT-symmetric microring resonators. Laser Photonics Rev..

[19] Karabchevsky A., Katiyi A., Ang A.S., Hazan A. (2020). On-chip nanophotonics and future challenges. Nanophotonics.

[20] Wiersig J. (2020). Review of exceptional point-based sensors. Photonics Res..

[21] Heiss W. (2000). Repulsion of resonance states and exceptional points. Phys. Rev. E.

[22] Chen W., Kaya Özdemir Ş., Zhao G., Wiersig J., Yang L. (2017). Exceptional points enhance sensing in an optical microcavity. Nature.

[23] De Carlo M., De Leonardis F., Soref R.A., Passaro V.M. (2022). Design of a trap-assisted exceptional-surface-enhanced silicon-on-insulator particle sensor. J. Light. Technol..

[24] Jiang S., Xiao Z., Li W., Chen T., Li J., Huang A., Zhang H. (2022). Enhanced nanoparticle sensing by mode intensity in a non-reciprocally coupled microcavity. J. Appl. Phys..

[25] Zhong Q., Ren J., Khajavikhan M., Christodoulides D.N., Özdemir Ş., El-Ganainy R. (2019). Sensing with exceptional surfaces in order to combine sensitivity with robustness. Phys. Rev. Lett..

[26] Liao Z., Peng X., Liu L., Xu Y., Xu K.D., Pan B., Luo G.Q., Liu Y. (2023). Microwave Plasmonic Exceptional Points for Enhanced Sensing. Laser Photonics Rev..

[27] Mao W., Fu Z., Li Y., Li F., Yang L. (2024). Exceptional–point–enhanced phase sensing. Sci. Adv..

[28] Tao Y., Liu W., Wang S., Nan C., Liu L., Bai Y., Zhou Y., Xing E., Tang J., Liu J. (2024). Ultra-stable control near the EP in non-Hermitian systems and high-precision angular rate sensing applications. Opt. Express.

[29] De Carlo M. (2021). Exceptional points of parity-time-and anti-parity-time-symmetric devices for refractive index and absorption-based sensing. Results Opt..

[30] Chaudhary P., Mishra A.K. (2023). Refractive index sensing near exceptional point of a system of triple microcavity. Sens. Actuators A Phys..

[31] Li Y., Deng Z., Qin C., Wan S., Lv B., Guan C., Yang J., Zhang S., Shi J. (2023). Bifunctional sensing based on an exceptional point with bilayer metasurfaces. Opt. Express.

[32] Jiang H., Zhang W., Lu G., Ye L., Lin H., Tang J., Xue Z., Li Z., Xu H., Gong Q. (2022). Exceptional points and enhanced nanoscale sensing with a plasmon-exciton hybrid system. Photonics Res..

[33] Feng L., Xu Y.L., Fegadolli W.S., Lu M.H., Oliveira J.E., Almeida V.R., Chen Y.F., Scherer A. (2013). Experimental demonstration of a unidirectional reflectionless parity-time metamaterial at optical frequencies. Nat. Mater..

[34] Lee H., Kecebas A., Wang F., Chang L., Özdemir S.K., Gu T. (2023). Chiral exceptional point and coherent suppression of backscattering in silicon microring with low loss Mie scatterer. eLight.

[35] Li J., Li W., Feng Y., Wang J., Yao Y., Sun Y., Zou Y., Wang J., He F., Duan J. (2024). On-Chip Fabrication-Tolerant Exceptional Points Based on Dual-Scatterer Engineering. Nano Lett..

[36] Zhen W., Ren Z.C., Wang X.L., Ding J., Wang H.T. (2025). Polarization structure transition of C-point singularities upon reflection. Sci. China Phys. Mech. Astron..

[37] Zhang J., Zhang L., Xu W. (2012). Surface plasmon polaritons: Physics and applications. J. Phys. D Appl. Phys..

[38] Gramotnev D.K., Bozhevolnyi S.I. (2010). Plasmonics beyond the diffraction limit. Nat. Photonics.

[39] Lee C., Lawrie B., Pooser R., Lee K.G., Rockstuhl C., Tame M. (2021). Quantum plasmonic sensors. Chem. Rev..

[40] Chin L.K., Son T., Hong J.S., Liu A.Q., Skog J., Castro C.M., Weissleder R., Lee H., Im H. (2020). Plasmonic sensors for extracellular vesicle analysis: From scientific development to translational research. ACS Nano.

[41] Xue T., Liang W., Li Y., Sun Y., Xiang Y., Zhang Y., Dai Z., Duo Y., Wu L., Qi K. (2019). Ultrasensitive detection of miRNA with an antimonene-based surface plasmon resonance sensor. Nat. Commun..

[42] Azzam S.I., Kildishev A.V., Ma R.M., Ning C.Z., Oulton R., Shalaev V.M., Stockman M.I., Xu J.L., Zhang X. (2020). Ten years of spasers and plasmonic nanolasers. Light Sci. Appl..

[43] Cho S., Yang Y., Soljačić M., Yun S.H. (2021). Submicrometer perovskite plasmonic lasers at room temperature. Sci. Adv..

[44] Liang Y., Li C., Huang Y.Z., Zhang Q. (2020). Plasmonic nanolasers in on-chip light sources: Prospects and challenges. ACS Nano.

[45] Balaur E., Cadenazzi G.A., Anthony N., Spurling A., Hanssen E., Orian J., Nugent K.A., Parker B.S., Abbey B. (2021). Plasmon-induced enhancement of ptychographic phase microscopy via sub-surface nanoaperture arrays. Nat. Photonics.

[46] Han X.X., Rodriguez R.S., Haynes C.L., Ozaki Y., Zhao B. (2021). Surface-enhanced Raman spectroscopy. Nat. Rev. Methods Prim..

[47] Stiles P.L., Dieringer J.A., Shah N.C., Van Duyne R.P. (2008). Surface-enhanced Raman spectroscopy. Annu. Rev. Anal. Chem..

[48] Zhang H.C., Cui T.J., Zhang Q., Fan Y., Fu X. (2015). Breaking the challenge of signal integrity using time-domain spoof surface plasmon polaritons. ACS Photonics.

[49] Dong J., Tomasino A., Balistreri G., You P., Vorobiov A., Charette É., Le Drogoff B., Chaker M., Yurtsever A., Stivala S. (2022). Versatile metal-wire waveguides for broadband terahertz signal processing and multiplexing. Nat. Commun..

[50] Haffner C., Chelladurai D., Fedoryshyn Y., Josten A., Baeuerle B., Heni W., Watanabe T., Cui T., Cheng B., Saha S. (2018). Low-loss plasmon-assisted electro-optic modulator. Nature.

[51] Chen J., Li Z., Yue S., Gong Q. (2010). Efficient unidirectional generation of surface plasmon polaritons with asymmetric single-nanoslit. Appl. Phys. Lett..

[52] Jin J., Li X., Guo Y., Pu M., Gao P., Ma X., Luo X. (2019). Polarization-controlled unidirectional excitation of surface plasmon polaritons utilizing catenary apertures. Nanoscale.

[53] Yang J., Zhou S., Hu C., Zhang W., Xiao X., Zhang J. (2014). Broadband spin-controlled surface plasmon polariton launching and radiation via L-shaped optical slot nanoantennas. Laser Photonics Rev..

[54] Zeng S.J., Zhang Q., Zhang X.M., Liu X.L., Xiao J.J. (2018). Unidirectional excitation of plasmonic waves via a multilayered metal-dielectric-metal Huygens’ nanoantenna. Opt. Lett..

[55] Yang X., Wang J., Lim X.H., Xu Z., Teng J., Zhang D.H. (2016). Unidirectional generation of surface plasmon polaritons by a single right-angled trapezoid metallic nanoslit. J. Phys. D Appl. Phys..

[56] Huang L., Wu S., Wang Y., Ma X., Deng H., Wang S., Lu Y., Li C., Li T. (2017). Tunable unidirectional surface plasmon polariton launcher utilizing a graphene-based single asymmetric nanoantenna. Opt. Mater. Express.

[57] Tyagi D., Chen T.Y., Huang C.B. (2020). Polarization-Enabled Steering of Surface Plasmons Using Crossed Reciprocal Nanoantennas. Laser Photonics Rev..

[58] Pors A., Nielsen M.G., Bernardin T., Weeber J.C., Bozhevolnyi S.I. (2014). Efficient unidirectional polarization-controlled excitation of surface plasmon polaritons. Light Sci. Appl..

[59] Huang L., Chen X., Bai B., Tan Q., Jin G., Zentgraf T., Zhang S. (2013). Helicity dependent directional surface plasmon polariton excitation using a metasurface with interfacial phase discontinuity. Light Sci. Appl..

[60] Lin J., Mueller J.B., Wang Q., Yuan G., Antoniou N., Yuan X.C., Capasso F. (2013). Polarization-controlled tunable directional coupling of surface plasmon polaritons. Science.

[61] Yang F., Mei Z.L. (2015). Guiding SPPs with PT-symmetry. Sci. Rep..

[62] Li Z.T., Li X., Liu G.D., Wang L.L., Lin Q. (2023). Analytical investigation of unidirectional reflectionless phenomenon near the exceptional points in graphene plasmonic system. Opt. Express.

[63] Wang W., Wang L.Q., Xue R.D., Chen H.L., Guo R.P., Liu Y., Chen J. (2017). Unidirectional excitation of radiative-loss-free surface plasmon polaritons in PT-symmetric systems. Phys. Rev. Lett..

[64] Xu Y., Li L., Jeong H., Kim S., Kim I., Rho J., Liu Y. (2023). Subwavelength control of light transport at the exceptional point by non-Hermitian metagratings. Sci. Adv..

[65] Zheng S., Yu W., Zhang W. (2022). Design and optimization of a passive PT-symmetric grating with asymmetric reflection and diffraction. Opt. Express.

[66] Özdemir Ş.K., Rotter S., Nori F., Yang L. (2019). Parity–time symmetry and exceptional points in photonics. Nat. Mater..

[67] Zayats A.V., Smolyaninov I.I. (2003). Near-field photonics: Surface plasmon polaritons and localized surface plasmons. J. Opt. A Pure Appl. Opt..

[68] Schinke C., Christian Peest P., Schmidt J., Brendel R., Bothe K., Vogt M.R., Kröger I., Winter S., Schirmacher A., Lim S. (2015). Uncertainty analysis for the coefficient of band-to-band absorption of crystalline silicon. AIP Adv..

[69] Ciesielski A., Skowronski L., Pacuski W., Szoplik T. (2018). Permittivity of Ge, Te and Se thin films in the 200–1500 nm spectral range. Predicting the segregation effects in silver. Mater. Sci. Semicond. Process..

[70] Johnson P.B., Christy R.W. (1972). Optical constants of the noble metals. Phys. Rev. B.

[71] Garcia-Vidal F.J., Martin-Moreno L., Ebbesen T., Kuipers L. (2010). Light passing through subwavelength apertures. Rev. Mod. Phys..

